# New record of *Didymocorypha* Wood-Mason (Mantodea, Eremiaphilidae) from China, with description of a new high-altitude wingless mantis species in Asia

**DOI:** 10.3897/zookeys.922.47987

**Published:** 2020-03-25

**Authors:** Chao Wu, Chun-Xiang Liu

**Affiliations:** 1 Key Laboratory of the Zoological Systematics and Evolution, Institute of Zoology, Chinese Academy of Sciences, Beichen West Road, Chaoyang District, Beijing 100101, China Institute of Zoology, Chinese Academy of Sciences Beijing China

**Keywords:** apterous mantis species, life history, new species, Oriental Region, taxonomy

## Abstract

The genus *Didymocorypha* Wood-Mason, 1877 (Eremiaphilidae, Iridinae) has only been recorded in South Asia, including a sole species *D. lanceolata* (Fabricius). Here, we firstly extend its distribution to China, with description of one new species *D. libaii***sp. nov.***Didymocorypha libaii***sp. nov.** lives in an area about 3000 meters above sea level on the southern slope of the Himalayas (Tibet in China), one of the highest-altitude inhabited areas of mantis in the Northern Hemisphere. It is also the first recorded Oriental mantis species in which both sexes are wingless. Life history of the new species, necessary illustrations and ecological images are provided. The distribution of the new *Didymocorypha* species is discussed and mapped.

## Introduction

The genus *Didymocorypha* was erected for a sole species *D. ensifera* Wood-Mason, 1877 from Sri Lanka with which *Pyrgocotis gracilipes* Stål, 1877 was synonymized (Wood-Mason 1882). Subsequently, *Mantis lanceolata* Fabricius, 1798, which was recorded from Eastern India, was transferred into the genus *Didymocorypha* by Bolivar (1897). Afterwards, *D. ensifera* was considered as a synonym of *D. lanceolata* (Kirby 1904). At the time of Ehrmann’s catalogue (Ehrmann 2002) the genus *Didymocorypha* only possessed one species, *D. lanceolata*, which is widely distributed in South Asia (e.g., Sri Lanka, Nepal and India). In Schwarz and Roy’s (2019) new taxonomic system, the genus *Didymocorypha* belongs to subfamily Iridinae (Eremiaphilidae). This subfamily includes eight genera, among which seven range from Africa to South Asia and one genus *Iris* Saussure ranges from Africa and Europe to northwestern China (Wang 1993). Until now, no other genera of the subfamily Iridinae or the family Eremiaphilidae have been recorded from China.

Within the order Mantodea, brachypterous females are common. Apterous females are a rarity except in some families, for example Thespidae and Haaniidae. It is also rare that both sexes of a certain species are wingless. As far as we know, both sexes are apterous in three Old World mantis genera, *Apteromantis* Werner, 1931 (Amelidae), *Geomantis* Pantel, 1896 (Rivetinidae), *Holaptilon* Beier, 1964 (Gonypetidae), one African genus *Apterocorypha* Roy, 1966 (Hoplocoryphidae) and one North American genus *Yersiniops* Hebard, 1931 (Amelidae) (Ehrmann 2002; Battiston et al. 2010). Most of these apterous mantis species are small-sized, and live in grassland or shrubland in temperate regions. No mantis species with apterous males and females has been recorded within the family Eremiaphilidae or in the Oriental Realm.

When investigating fauna on the southern slope of the Himalayas in Tibet, China, we collected apterous adult specimens of *Didymocorypha* from Gyirong County at an altitude of 3000 meters in 2017. After dissecting the male specimens and comparing them with Indian samples of *D. lanceolata*, we thought that those wingless specimens should belong to a unique new species of *Didymocorypha*. The new species is the first recorded species of *Didymocorypha* from China, and the first recorded Oriental mantis species with wingless male and female adults. *Didymocorypha* is the second recorded genus of the family Eremiaphilidae from China. Here, we review the genus *Didymocorypha*, provide a redescription of the known species, and thoroughly describe the new species and its life history.

## Material and methods

Classification system follows Schwarz and Roy (2019). Descriptive terminology of adult morphology and the male genitalia follows Brannoch et al. (2017) and Schwarz and Roy (2019). All specimens of the new species were collected during daytime through careful observation. Genitalia were dissected in 10% KOH solution, cleared with pure water, and finally stored in 70% ethanol in Eppendorf tubes for further research. Pictures were taken with a Nikon digital camera.

The specimens were deposited in the following institutions or private collections.

**IZCAS** Institute of Zoology, Chinese Academy of Sciences, Beijing, China

**CJZ** Collection of Jia-Zhi Zhang, Shanghai, China

**CWC** Collection of Chao Wu, Beijing, China

## Taxonomic treatment

### Didymocorypha

Taxon classificationAnimaliaMantodeaEremiaphilidae

Wood-Mason, 1877

30FFF2D2-F934-5D1F-8909-042C8E2245E1

**Figure 1.**
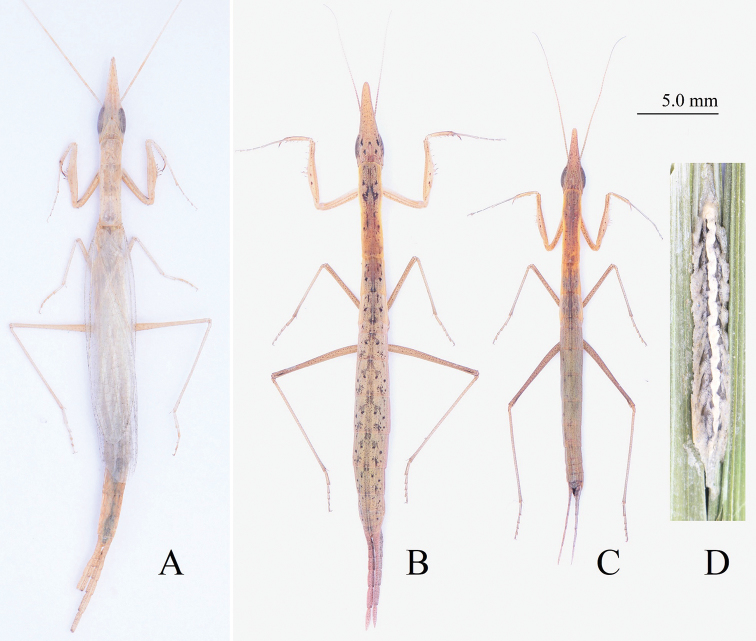
*Didymocorypha* spp. body in dorsal view and ootheca. **A, C** Male **B** female **D** oothecae. **A***D. lanceolata* (Fabricius) **B–D***D. libaii* sp. nov. (holotype and paratype).

**Figure 2.**
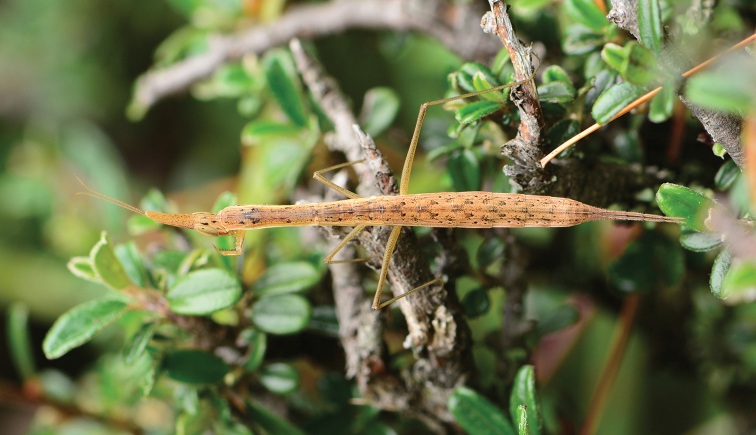
*Didymocorypha libaii* sp. nov. adult female in its natural habitat.

**Figure 3.**
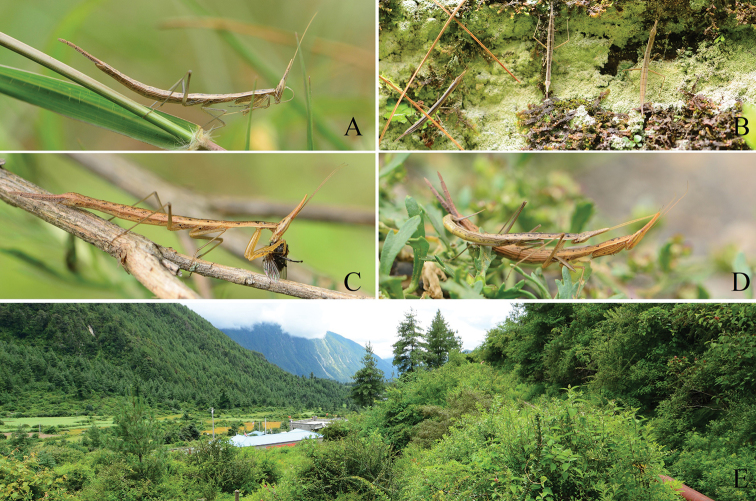
Adult and nymph of *Didymocorypha libaii* sp. nov. in natural habitat. **A** Adult male **B** nymphs **C** feeding adult female **D** copulating adults **E** ecological habitat.

**Figure 4.**
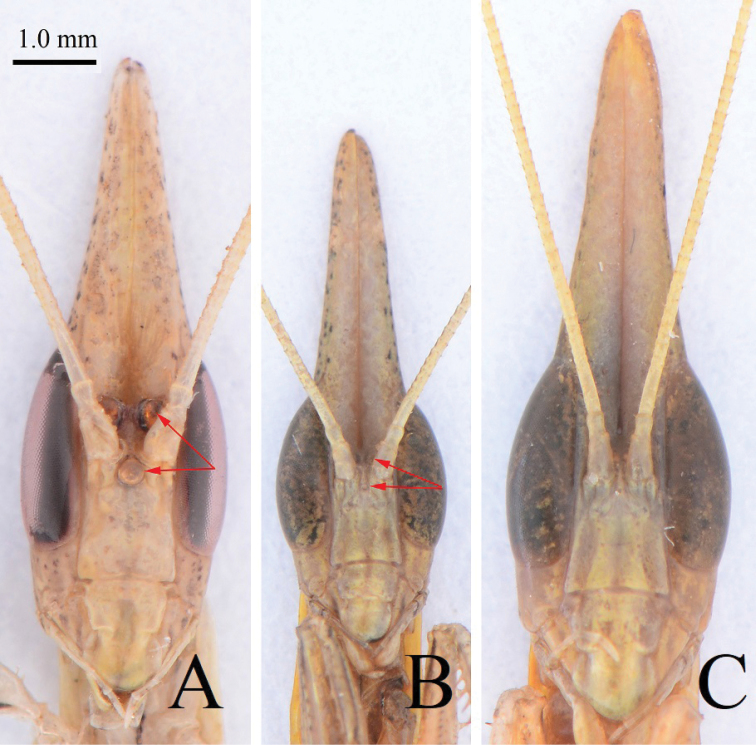
Head of *Didymocorypha* spp., anterior view. **A** Male, *D. lanceolata* (Fabricius) **B** male, *D. libaii* sp. nov. (holotype) **C** female, *D. libaii* sp. nov. (paratype). Red arrows point to ocelli.

**Figure 5.**
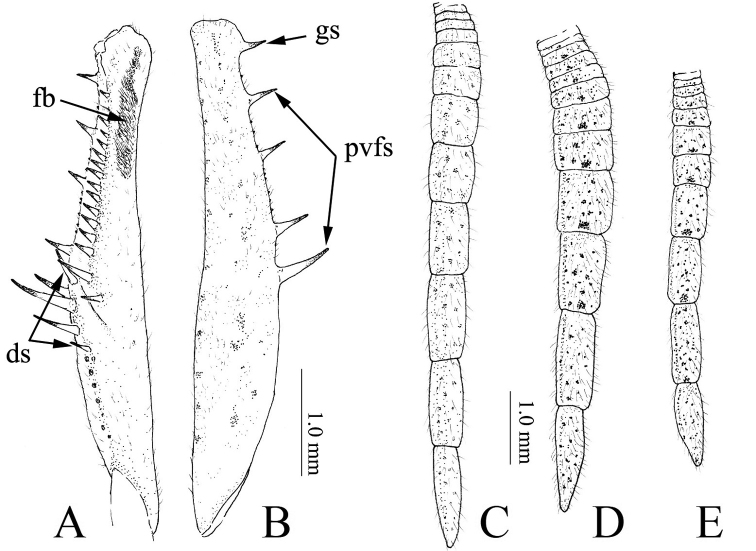
Fore femur (**A, B**) and cerci (**C–E**) of *Didymocorypha* spp. **A, B, D, E***D. libaii* sp. nov. **C***D. lanceolata* (Fabricius). **A** Ventral view **B** dorsal view **A, B, C, E** male **D** female. Abbreviations: **fb** = femoral brush; **ds** = discoidal spines; **gs** = genicular spur; **pvfs** = posteroventral femoral spines.

**Figure 6.**
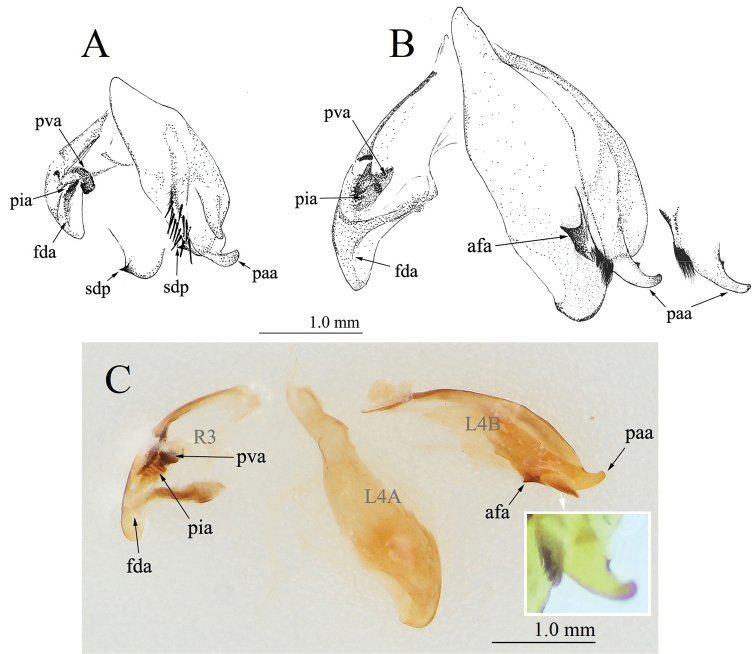
Male genitalia of *Didymocorypha* spp., Disarticulated genital complex, ventral view. **A***D. lanceolata* (Fabricius) **B, C***D. libaii* sp. nov. Abbreviations: **L4A** = sclerite extending over the ventral wall of left phallomere; **L4B** = sclerite extending over the dorsal wall of left phallomere; **R3** = anteriorly extending sclerite of right phallomere; **afa** = phalloid apophysis; **fda** = main posterior lobe of right phallomere; **pia** = process posterolateral to pva of right phallomere; **pva** = process anteromesal to pia of right phallomere; **paa** = posterior process of left phallomere; **sdp** = secondary distal process.

**Figure 7.**
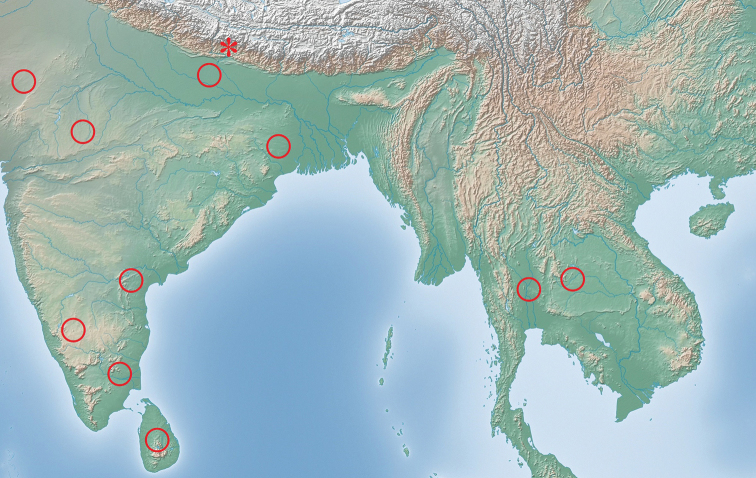
Distribution map of the distribution *Didymocorypha* spp. in South Asia and East Asia (Himalaya).○: *D. lanceolata* (Fabricius) ; * *D. libaii* sp. nov.

Figs 1 , 2 , 3 , 4 , 5 , 6 , 7

Schizocephalus (Didymocorypha) : Wood-Mason, 1877: 221.
Pyrgocotis : Stål, 1877: 14; Westwood 1889: 3; Brunner de Wattenwyl 1893: 59; Kirby 1904: 218 (syn.); Giglio-Tos 1921: 31 (syn.).
Didymocorypha : Wood-Mason, 1882: 24; Westwood 1889: 3; Brunner de Wattenwyl 1893: 59; Bolivar 1897: 303; Kirby 1904: 218; Giglio-Tos 1919: 57; Giglio-Tos 1921: 31; Giglio-Tos 1927: 116; Beier 1935: 5; Beier 1964: 942; Beier 1968: 8; Ehrmann 2002: 122; Otte and Spearman 2005: 328; Ehrmann and Borer 2015: 231; Schwarz and Roy 2019: 115, 143.

#### Type species.

Schizocephalus (Didymocorypha) ensifera Wood-Mason, 1877 by original monotypy =*Mantis lanceolata* Fabricius, 1798.

#### Diagnosis.

Small-sized, slender (Figs 1–3). Head elongate (Fig. 4), with lateral lobes of vertex prolonged into triangular processes, running alongside each other but not fused. Compound eyes large, oblong. Lower frons approximately trapezoid. Pronotum slender, with nearly parallel lateral margins. Fore legs weak. Fore femur (Fig. 5A, B) with 4 ventro-posterior and 4 discoidal spines; claw groove in the middle; fore tarsus much longer than tibia, and basal tarsomere longer than total length of remaining segments. Middle and hind legs slim without expansions but with genicular spines. Hind legs longer and stronger than mesolegs, similar to jumping legs of locusts. Male winged (Fig. 1A) or wingless (Figs 1C, 3A); if winged, fore wings hyaline, a little shorter than body. Female wingless (Figs 1B, 2, 3C).

Abdomen long, narrow. Cerci well-developed, with each segment wide, flat, lanceolate (Fig. 5C–E).

#### Distribution

(Fig. 7). India, Nepal, Sri Lanka, China (new record).

### Didymocorypha lanceolata

Taxon classificationAnimaliaMantodeaEremiaphilidae

(Fabricius, 1798)

56E154DA-010B-59FB-B3D3-CCAC57136CA7

Figs 1A , 4A , 5C , 6A , 7

Mantis lanceolata : Fabricius, 1798: 191.
Schizocephalus (Didymocorypha) ensifera Wood-Mason, 1877: 221–222.
Pyrgocotis gracilipes : Stål, 1877: 17; Westwood 1889: 3 (syn.); Kirby 1904: 218 (syn.); Giglio-Tos 1927: 116.
Didymocorypha ensifera : Wood-Mason, 1882: 24; Wood-Mason 1889: 34; Kirby 1904: 218 (syn.); Giglio-Tos 1927: 116.
Pyrgomantis lanceolata : Westwood 1889: 3.
Didymocorypha lanceolata : Bolivar 1897: 303; Kirby 1904: 218; Giglio-Tos 1921: 32; Giglio-Tos 1927: 116; Henry 1932: 9; Werner 1933: 898; Ehrmann 2002:123; Otte and Spearman 2005: 328; Ehrmann and Borer 2015:231, 244, 249; Schwarz et al. 2018: 206–207, 227.

#### Type locality.

‘India orientali’ (Fabricius 1798).

#### Material examined.

India • 5 ♂; Andhra Pradesh, Nellore District; 15.769N, 79.693E; 150 m; 10~25-IX-2012; IZCAS.

#### Description.

**Male.** Slim and slender, withered-grass-like (Fig. 1A). Three ocelli large and hump (Fig. 4A). Fore femur approximately as long as fore coxa, with 4 posteroventral, 4 discoidal, 17 anteroventral spines; claw groove lying basally than middle of fore femur. Fore tibia about half as long as femur, with 5 posteroventral, 10 anteroventral spines and 1 strong tibial spur. Wings hyaline and iridescent, a little shorter than body; fore wings long and narrow, hind wings broad. Cerci flat, wide, lanceolate, with distal joints gradually becoming longer distad (Fig. 5C).

External genitalia (Fig. 6A) small; left phallomere narrow, long, with finger-like process paa and about 12 thick bristles on the afa; ventral phallomere with a robust short sharp spd.

**Female** similar as male, but larger, stronger, and wingless.

***Male measurements*** (Length in mm). Body: 34.60–35.05; head: 7.10–7.14; pronotum: 5.90–5.95; fore coxae: 2.90–2.95; fore femora: 3.18–3.22; fore tibiae: 2.39–2.41; middle femora: 3.57–3.60; hind femora: 6.65–6.70; forewing: 14.05–14.10; hind wing: 15.33–15.38; cercus: 8.70–8.75.

#### Distribution

(Fig. 7). India, Nepal, Sri Lanka (Ehrmann 2002), Thailand (Unnahachote et al. 2019).

### Didymocorypha libaii

Taxon classificationAnimaliaMantodeaEremiaphilidae

Wu & Liu sp. nov.

AD5C9BD4-CD4B-59F4-A036-EF091ECF1376

http://zoobank.org/B5D329E2-4E92-4853-911E-C6753EE240F3

Figs 1B–D , 2 , 3 –D, 4B, C, 5A, B, 5D, E, 6B, C, 7

#### Material examined.

***Holotype*.** China • ♂; Tibet, Gyirong County; 28.404N, 85.332E; 3300 m; 20-VII-2017; Chao Wu leg.; IZCAS. ***Paratypes*.** China • 4 ♂ 6 ♀; Tibet, Gyirong County; 28.397N, 85.351E; 2800~3300 m; 18~21-VII-2017; Chao Wu leg.; IZCAS • 3 ♂ 3 ♀; ditto; CWC • 1♀; ditto; CJZ • 1 ♀; Tibet, Gyirong County; 28.363N, 85.339E; 2672 m; 1-VIII-2018; Jin-Cheng Liu leg.; CWC.

#### Description.

Holotype. **Male.** Slim (Figs 1B, C, 2, 3A, 3C).

***Head***: lanceolate. Paired juxtaocular bulges united into a conical extension with a complete median dorsal suture and a deep vertical ventral groove (Fig. 4B). Compound eyes long, oval, not bulging. Three ocelli, small, not obvious (Fig. 4B). Lower frons approximately trapezoidal, approximately as wide as high.

***Thorax***: pronotum longer than head, slender, about 3 times as long as wide. Prozona almost as wide as metazona. Mesothorax similar to metathorax, simple, nearly trapezoidal. Thorax with distinct medial keel. Wingless.

***Prothoracic legs***: fore coxa smooth, unarmed, shorter than metazona; fore femur as long as coxa, with a strongly-developed genicular spur (Fig. 5B), 4 posteroventral, 4 discoidal, 15–16 anteroventral spines, and without dilation on dorsal surface (Fig. 5A,B); claw groove lying basally to middle of fore femur; fore tibia about half as long as femur, with 5–6 posteroventral, 10 anteroventral tibial spines and 1 strong tibial spur; fore tarsus longer than tibia; basal tarsomere (= basitarsus) longer than total length of remaining segments.

***Meso- and metathoracic legs***: slim without expansions and with one small femoral genicular spur and one obvious tibial spur. Tarsus much shorter than tibia; basal tarsomere short, less than total length of remaining segments. Metathoracic legs longer and stronger than mesolegs.

***Abdomen***: almost as wide as pronotum. Each abdominal segment similar, nearly square; tergite 10 (Supra-anal plate) broad, widely trianglar. Cerci possessing 15 joints, with distal joints gradually becoming longer distad. Each of last 3 joints longer than wide (Fig. 5E). Coxosternite 9 (subgenital plate) nearly triangular, slightly asymmetrical, with a pair of styli.

***External genitalia*** (Fig. 6B, C): relatively large-sized. Left phallomere narrow and long, posterior process of ventral phallomere (spd) indistinct; phalloid apophysis (afa) short, wide and strongly sclerotized, with a spine-like projection; posterior process of left phallomere (paa) with a finger-like extension, with a small obtuse tubercle in middle, and with a brush-like cluster of hairs on base.

**Female.** Similar to male, but distinctly larger and stronger (Figs 1B, 5C).

***Measurements*** (Length in mm, Holotype in parentheses). Body: male 28.30–28.75 (28.45), female 32.50–35.15; head: male 5.85–5.95 (5.94), female 7.45–7.55; pronotum: male 5.35–5.39 (5.39), female 6.95–7.10; fore coxae: male 3.13–3.18 (3.15), female 4.11–4.20; fore femora: male 4.10–4.13 (4.11), female 4.62–4.80; fore tibiae: male 2.25–2.30 (2.27), female 2.85–3.02; middle femora: male 4.42–4.51 (4.45), female 5.70–5.79; hind femora: male 6.20–6.27 (6.25), female 7.43–7.52; cercus: male 5.45–5.50 (5.47), female 7.30–7.35.

#### Diagnosis.

The new species is distinguished from *D. lanceolata* by small body size, small and indistinct male ocelli, wingless male adults, comparatively large-sized genitalia, ventral phallomere without secondary distal process (sdp), additional obtuse tubercle on paa and different structure of afa (Fig. 6).

#### Coloration

(Figs 2, 3). Monotonous, tawny, dry-grass-like, densely covered with little black spots. Some specimens possessing irregular black patches. Spines of fore legs brown.

#### Life history.

The new species often lives at the bottom of bushes in a variety of angiosperms (Figs 2, 3A–D) in high-altitude coniferous forest. Nymphs were found to be clustering (Fig. 3B), without cannibalism. This peaceful situation is an exception for mantis. The mating (Fig. 3D) is also peaceful, and needs up to 4–8 hours. Female lays their oothecae on the fifth day after mating. Oothecae are fusiform, withered-leaf-like. Each ootheca contains 4–10 eggs (Fig. 1D). Color of ootheca varies from light to very dark brown. External wall of cotheca is thin, sparse.Oothecae did not hatch successfully in the laboratory probably due to significant elevation differences from the mantis’s natural habitat. In field, the mantis species prey on small-sized insects (e.g., Diptera, Hemiptera and Collembola) (Fig. 3C), based on our observations.

#### Distribution.

China (Tibet: Gyirong County).

#### Etymology.

The new species was named after Bai Li, who is a poet in the Tang dynasty of China and one of the most famous poets in Chinese history.

## Discussion

*Didymocorypha libaii* sp. nov., is the first mantis species recorded at altitudes of more than 3000 meters (Fig. 3E) in China. At the type locality of *D. libaii* sp. nov., blankets of snow persist during the long winter, and the growing period is very short. In fact, it was difficult to distinguish the adults and nymphs of this new species from each other in general appearance. Initially, we judged them to be adults because they were mating when breeding indoors. Retention of nymph characteristics in the adults is called neoteny. We assume in the harsh environment of type locality of *D. libaii* sp. nov., that neotenic development could help to shorten the life cycle of the mantis, simultaneously, the large-sized male genitalia of the species can improve the success rate of copulation. In summary, the wingless adults and the large-sized male genitalia enable the species to adapt to the harsh environment.

We suppose that the new species was isolated by the uplifted Himalayas and diverged from its congener. Its ancestral population adapted to the environment at high altitudes, and was restricted to a very narrow range. In addition, a few mantis species (of genera *Arria* Stål, 1877, *Odontomantis* Saussure, 1871 and *Phyllothelys* Wood-Mason, 1877) are also found at an altitude of about 2500 m in China (including high-altitude areas of the Himalayas) based on our collections, which we will report in other papers. There are a range of suitable environments on the southern slopes of the Himalayas in China and more discoveries will possibly be made in the future. The other recorded mantis species at high altitude include *Pseudopogonogaster hebardi* (Terra, 1982) from Ecuador at elevations 3500 m and *Armene breviptera* Lindt, 1963 from Badakhshan (West Pamir Mountains) at elevations 2300–2700 m. One ootheca of *A. breviptera* was even found at 3700 m (Lindt 1963). *Armene breviptera* was the only species of Mantodea that was previously found in the harsh environment. The dominant ecosystem there is dry mountain grassland with short and sparse vegetation cover, without trees or bushes and with very low biodiversity comparing to the lower elevations in the same region. The snow cover during winter is intermittent and often does not provide sufficient thermal protection during the cold months (Khakimov et al. 2007). *Armene breviptera* is the only micropterous species in the genus, also suggesting a connection between harsh external conditions and wing adaptation in Mantodea. By comparison, *Didymocorypha libaii* sp. nov. lives in a significantly milder environment with abundant vegetation, including trees, in spite of long winters. The conditions of *D. libaii* are also atypical for Mantodea also suggesting possible adaptations of the species to the short growth period.

## Supplementary Material

XML Treatment for Didymocorypha

XML Treatment for Didymocorypha lanceolata

XML Treatment for Didymocorypha libaii
